# Remediation Agents Drive Bacterial Community in a Cd-Contaminated Soil

**DOI:** 10.3390/toxics11010053

**Published:** 2023-01-04

**Authors:** Wenzhi Cui, Yingying Liu, Wenguang Li, Lei Pei, Shuang Xu, Yuhuan Sun, Jianbo Liu, Fayuan Wang

**Affiliations:** College of Environment and Safety Engineering, Qingdao University of Science and Technology, Qingdao 266042, China

**Keywords:** soil amendments, cadmium, soil remediation, soil microbial diversity, marker species

## Abstract

Soil remediation agents (SRAs) such as biochar and hydroxyapatite (HAP) have shown a promising prospect in in situ soil remediation programs and safe crop production. However, the effects of SRAs on soil microbial communities still remain unclear, particularly under field conditions. Here, a field case study was conducted to compare the effects of biochar and HAP on soil bacterial communities in a slightly Cd-contaminated farmland grown with sweet sorghum of different planting densities. We found that both biochar and HAP decreased the diversity and richness of soil bacteria, but they differently altered bacterial community structure. Biochar decreased Chao1 (−7.3%), Observed_species (−8.6%), and Shannon indexes (−1.3%), and HAP caused Shannon (−2.0%) and Simpson indexes (−0.1%) to decline. The relative abundance (RA) of some specific taxa and marker species was differently changed by biochar and HAP. Overall, sweet sorghum cultivation did not significantly alter soil bacterial diversity and richness but caused changes in the RA of some taxa. Some significant correlations were observed between soil properties and bacterial abundance. In conclusion, soil remediation with biochar and HAP caused alterations in soil bacterial communities. Our findings help to understand the ecological impacts of SRAs in soil remediation programs.

## 1. Introduction

Soil contamination with toxic metals such as cadmium (Cd) poses a serious risk to food safety [1,2] and human health [3]. The use of soil remediation agents (SRAs) for in situ stabilization provides a feasible solution to soil Cd pollution. Biochar [4,5] and hydroxyapatite (HAP) [6,7,8] are two common SRAs for in situ stabilization, which can reduce the transfer of toxic metals to food chains by different mechanisms [9]. Biochar is a solid material obtained from the thermochemical conversion of biomass in an oxygen-limited environment. Porous structure and abundant oxygen-containing functional groups in the biochar can stabilize heavy metals in the soil through physical sorption, precipitation, complexation, ion exchange, and electrostatic interaction [5,10,11]. Similarly, HAP can stabilize heavy metals via ion exchange, surface complexation, and the formation of phosphate precipitates [12,13,14,15]. By employing these immobilization mechanisms, biochar and HAP can reduce the availability of heavy metals in the soil and the uptake of heavy metals by plants, in particular crops, thus decreasing the entry of the metals into food chains.

The application of SRAs in soil remediation may change soil microbial communities directly and indirectly. First, some SRAs can provide carbon sources and nutrients for microbes. One case is biochar, as a carbon source, which can provide an amount of labile carbon for microorganisms [16]. Previous studies have found that the labile carbon carried by biochar acts as a carbon source for specific soil bacteria [17], such as *Gemmatimonas* [18,19,20,21] and *Bacillus* [22]. Second, SRAs such as biochar and HAP can change soil physicochemical properties, such as pH and dissolved organic carbon, thus indirectly affecting microbial community structure and diversity [8,16,23]. For example, biochar may improve the habitat of Gemmatimonadetes by reducing soil water fluctuations, because Gemmatimonadetes are unable to resist moisture fluctuations induced by wet/dry cycling [24]. HAP can increase soil pH and soluble phosphate [7,25], which can affect soil microbial communities, such as Gammaproteobacteria and Xanthomonadales [8]. Furthermore, SRAs generally decrease the bioavailability of toxic pollutants in soil [8,26], which may alleviate their negative effects on microbial communities. Considering their important ecological functions, the changes in soil microbial communities by SRAs during soil remediation processes should be evaluated.

However, microbial community structure usually varies with habitats and pollutants [27]. Although previous studies have explored the effects of SRAs on microbial communities in soil remediation processes [28,29,30,31], few studies compare the effects of different SRAs, particularly in field conditions. Plant density is recognized as one of the critical factors determining the efficiency of phytoremediation programs [32,33]. With these ideas in mind, we investigated the effects of biochar and HAP on soil bacterial communities in a slightly Cd-contaminated farmland soil with different planting densities. Sweet sorghum was selected as the test plant, because it can be used as either a forage or bioenergy crop to remediate polluted soil [34]. Seedlings meeting the quality standard can be used to feed livestock, while those with excess contaminants can be used to produce ethanol [35]. Here, we hypothesize that SRAs and planting densities can jointly affect soil properties and soil microbial communities. Thus, our aims are to elucidate: (1) whether SRAs (biochar and HAP) affect soil properties and Cd bioavailability in the soil with different planting densities, and (2) how soil bacterial communities respond to biochar and HAP.

## 2. Materials and Methods

### 2.1. Soil, Plants and Amendments

The field experiment was conducted on farmland slightly contaminated with Cd, located in Baoluo Village, Pingdu, Shandong Province, China (119°41′37.94″ E, 36°50′4.07″ N), which is situated near mine tailings from local gold and graphite mines. This area has a temperate monsoon climate, with an annual average temperature of 11.9 °C and annual average precipitation of 788.4 mm. During June to October, the monthly precipitation is 44~165 mm and the average maximum and minimum temperature is 20–28 and 13–23 °C, respectively. The soil texture is classified as sandy loam according to the USDA classification. Some soil properties are shown in Table 1.

The sweet sorghum cultivar was Hunnigreen. Two common agents were applied, including HAP and biochar. HAP was purchased from Sichuan Mianyang Xingheyi New Material Technology Co., Ltd. (Mianyang, Sichuan, China). HAP was an ultra-fine powder with pH of 7.5, Ca_10_(PO_4_)_6_OH_2_ content ≥ 99.6%, total Cd content 0.5 mg kg^−1^, and average particle size of 505 µm. SEM images are shown in Figure S1. Biochar was purchased from Lvzhiyuan Activated Carbon Co., Ltd. (Pingdingshan, China). It was generated by heating the straws of wheat and corn, wood chips, and fruit shells under a 500–600 °C limited-oxygen environment, with the following properties: pH 7.3, average specific surface area 863 m^2^ g^−1^, ash content 9%, moisture content 8.5%, methylene blue adsorption 6 mL (0.1 g)^−1^, iodine value 835 mg g^−1^, and average particle size ≤ 74 µm, As 1.4 mg kg^−1^, and Hg 0.4 mg kg^−1^. Pb, Cd, and Cr were not detected in the biochar.

### 2.2. Experimental Design and Sample Collection

The experiment included three SRA treatments (i.e., biochar treatment (B), HAP treatment (P), no SRAs (C)), each with three planting densities of sweet sorghum, i.e., high density (H), low density (L), and no planting (N). Each treatment was replicated in three plots (6 × 3 m). SRAs were manually applied in the plots at a rate of 2.78 t ha^−1^ and thoroughly mixed into the surface soil of 0–20 cm depth. Five kilograms of biochar or HAP was added to each plot. Plots were separated by 20 cm-high ridges to prevent soil and water exchange. Then, uniform seeds were selected and surface-sterilized, and then sown on 20 June 2021. After two weeks, the seedlings were thinned to a density of 35 × 20 cm in high-density treatment (marked as H) and a density of 60 × 30 cm in low-density treatment (marked as L). A treatment without planting was set as the control treatment (marked as N).

The seedlings were managed according to local agricultural practices. No fertilizers and pesticides were applied, but weeds were uprooted artificially and irrigation was performed when required during the growth stage. Manure plants were harvested on 26 October 2021. Ten plants were randomly selected from each plot, and leaves, stems, and roots were separated and taken back to measure the fresh and dry weights and Cd content. Ten soil samples were randomly collected at a depth of 0–20 cm from each plot and mixed thoroughly into one sample. Approximately 200 g soil samples were placed into sterile plastic bags in a frozen box and sent back immediately to the laboratory. About 5 g soil was placed into 5 mL cryopreservation tubes and frozen in −196 °C liquid nitrogen for soil bacteria analysis. The remaining samples were air-dried, ground, and used to analyze soil physicochemical properties and Cd.

### 2.3. Analysis of Soil and Plant Samples

Soil pH was measured at a water-to-soil ratio of 2.5:1 (*w*/*v*) using a pH meter (HJ 962-2018, China). Available phosphorus (AP) was determined by the molybdenum antimony anti-spectrophotometric method after extraction with a NH_4_F–HCl solution (NY/T 1121.7-2014, China). Available potassium (AK) was determined by Atomic Absorption Spectroscopy (AAS) (AA-7000, Shimadzu, Kyoto, Japan) after extraction with 1 M CH_3_COONH_4_ solution (NY/T 889-2004, China). NH_4_^+^-N and NO_3_^−^-N were determined by indophenol blue colorimetry and ultraviolet spectrophotometry after extraction with 2 M KCl [36]. Soil samples were digested using acid digestion (HCl-HNO_3_-HF-HClO_4_) to determine the concentrations of total metals (GB/T 17141-1997, China). Plant samples were digested in a HNO_3_–HClO_4_ (4:1, *v*/*v*) mixture in a Graphite Digestion Instrument (SH220N, Shandong Hanon Instruments Co., Ltd., Jinan, China). Soil available Cd, Cu, and Zn were extracted using DTPA solution (HJ 804-2016, China). Cd availability was also evaluated using the TCLP method [37]. The metal concentrations in the digested solution and extraction were determined using AAS. For analytical quality control, the blank and parallel samples were analyzed to verify the extraction process and reproducibility of analytical results. The recovery percentage ranges from 85% to 115%. The coefficient of variation of parallel samples is <20%. The quantification limit of AAS is 0.005 mg L^−1^.

### 2.4. Analysis of Soil Bacterial Communities

To analyze soil bacterial communities, soil samples were sent to Shanghai Personalbio Technology Co. Ltd. (Shanghai, China) to perform 16S rRNA sequencing. Briefly, DNA was extracted, and specific primers (F: ACTCCTACGGGAGGCAGCA R: CGGACTACHVGGGTWTCTAAT) were used to amplify the V3–V4 hypervariable region of bacterial 16S rRNA genes. Then, the DADA2 method [38] in the QIIME2 platform was used to carry out the steps of priming removal, mass filtering, denoise, stitching, and de-embedding, and the resulting sequences were named Amplicon Sequence Variants (ASVs). Finally, the representative sequence of each ASV was assigned to a taxonomic lineage based on the Silva database (Release132, http://www.arb-silva.de) [39], thereby taxonomic lineages composition data (bacterial relative abundance data) were generated. Phylogenetic trees were constructed by using FastTree in the maximum likelihood method. Ninety-five percent of the minimum sample sequence size was designed to sequencing depth for further analysis.

### 2.5. Data Analysis

Data were analyzed using SPSS 26.0 (IBM SPSS) to obtain the significance and variance. Duncan test and one-way ANOVA were employed to test the significance of the differences in edaphic factors and plant Cd content. Because this was a field experiment with many sources of uncontrolled variance, the minimum level of significance for assessing the results as worthy of note was 0.10, which means that there is a 10% probability that we will make a mistake when rejecting the hypothesis. Two-way ANOVA was used to compare the effects of interactions between amendments and sweet sorghum density.

Using the Personalbio platform (https://www.genescloud.cn/cloudClassroom), Pearson correlation (*p* < 0.05) was conducted to examine relationships between soil properties and soil bacterial communities. Diversity indexes were used to extract and calculate ASVs data, including the Chao1, Faith_pd, Pielou_e, Goods_coverage, Observed_species, Shannon, and Simpson diversity indices (http://scikit-bio.org/docs/latest/generated/skbio.diversity.alpha.html#module-skbio.diversity.alpha). Based on UPGMA and inter-group differences, the species heatmaps showed the differences in species composition. The differences in taxonomic lineages between groups were tested by the K–W test, and the clustering algorithm UPGMA was performed according to the Pearson correlation coefficient matrix of taxonomic lineages composition data (bacterial relative abundance data). MetagenomeSeq analysis was conducted using the fitFeatureModel function to fit the distribution of each ASV/OTU using the zero-vehicle log-normal model. Then, the up-regulation group and control group were selected to generate a Manhattan map. In addition, LEfSe analysis could remove the influence of other factors to find the most robust marker species in different soil treatment types. K–W and Wilcoxon rank sum tests were used to determine the differential species, and LDA analysis was performed to estimate the effect size of the abundance of each differential component (differential species) on the difference between groups. The differential species passing the threshold that was set up in LDA analysis could be considered biomarkers. According to the above results, the cladogram of LEfSe analysis and LDA histogram could be generated. PCoA was performed to determine the effect of SRAs on the microbial phylogenetic communities. The Bray–Curtis algorithm was used to calculate the distance between samples, and the ordination of all samples was obtained through multidimensional scaling. Then, regression fitting analysis between environmental factors and ordination was performed using regression function envfit, and significance (*p* value) was calculated using Permutation.

## 3. Results

### 3.1. Soil Properties and Cd Content in Plants

Two-way ANOVA results (Table S1) showed that SRA type significantly influenced soil pH (*p* < 0.1), AK (*p* < 0.01), DTPA-Cd (*p* < 0.01), and -Cu (*p* < 0.05), and planting density changed AK (*p* < 0.01), DTPA-Zn (*p* < 0.1), and NO_3_-N (*p* < 0.1). No significant interactive effects were observed between sweet sorghum density and SRA type (Table S1). The soil without planting and SRAs had the highest pH, AK, and DTPA-Zn (Table S2). Biochar and planting treatments had lower soil pH than the control and HAP treatments.

Overall, SRAs and planting density did not significantly change Cd concentration in plant roots and stems (Table S1). Compared to the control, biochar had no significant effects on DTPA-Cd concentration, but HAP treatments showed an increasing trend (Table S2). Cd was only detected in the leaves from the treatments of low planting density and no SRAs.

Pearson correlation analysis (Table S3) showed that DTPA-Cd was positively correlated with DTPA-Zn and -Cu (*p* < 0.05). Cd concentrations in roots and stems were positively correlated with soil DTPA-Cd (*p* < 0.001). Soil pH was positively correlated with AK (*p* < 0.05) and DTPA-Zn (*p* < 0.1).

### 3.2. Soil Bacterial Communities Structure and Diversity

After quality filtration, a total of 1,135,637 effective sequences (average sequence length 415 bp) were obtained from 27 soil samples (range 31,081–57,565 sequences per sample) (Table S5 and Figure S2). The ASVs per sample, ranging from 3383 to 4281, were identified by DADA2, and more than 60% of ASVs can be classified into genera (Figure S3).

**Figure 1:**
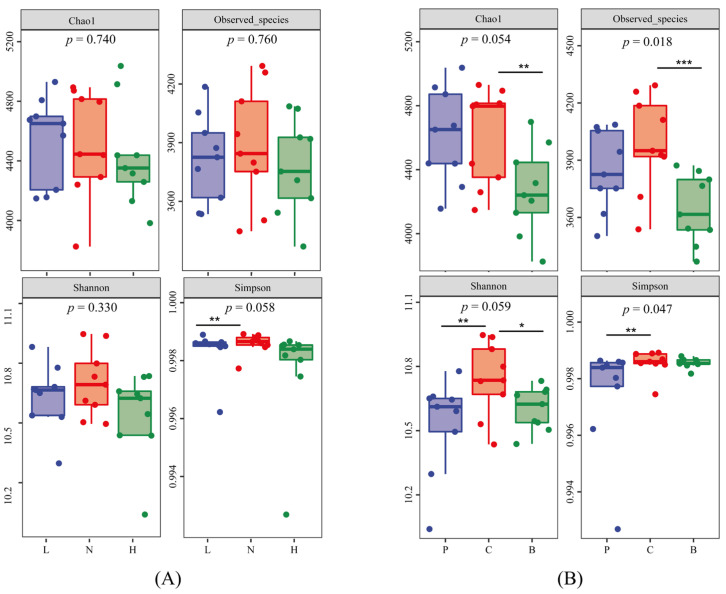
The α diversity of soil bacterial communities in the plots with different planting densities (**A**) and SRAs (**B**). *p*-values (* *p* < 0.1, ** *p* < 0.05, *** *p* < 0.01) were obtained from K–W tests. P, C, and B represent hydroxyapatite addition, no amendments, and biochar addition, respectively. L, N, and H represent low planting density, no planting, and high planting density, respectively.

Two-way ANOVA results showed that, in most cases, the α diversity of soil bacterial communities was not influenced by planting density (except Faith_pd index), but significantly changed by SARs (except Simpson index). As shown in Figure 1, biochar decreased the values of Chao1, Observed_species, and Shannon indexes, and HAP decreased Shannon and Simpson indexes. Low planting density caused a lower Simpson index.

Interactive effects on bacterial α diversity were observed between SARs and planting density (Table S1). For example, high planting density decreased Chao1 index singly or co-existing with biochar, but showed an increasing trend when co-existing with HAP (Figure S4). The treatment with biochar and high planting density had the lowest Chao1 index.

Pearson correlation analysis revealed the relationship between soil physicochemical properties and α-diversity index (Table S4). DTPA-Cd was negatively correlated with Shannon and Simpson, while AK, AP, and pH were positively correlated with Chao1 and Observed_species.

**Figure 2:**
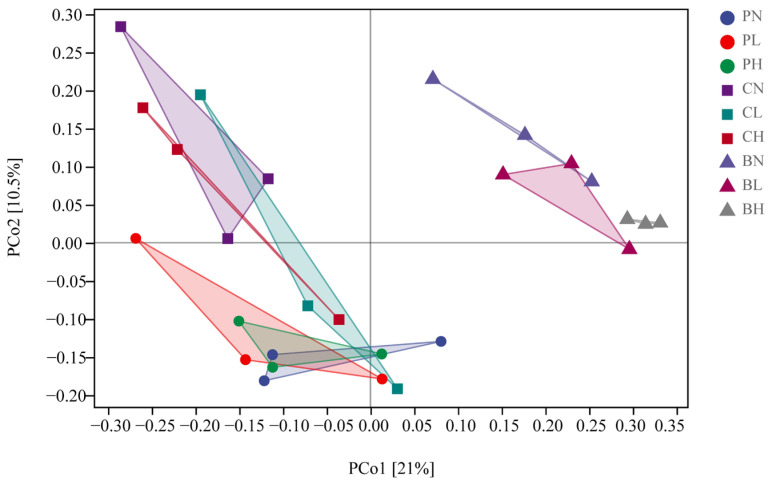
Principal coordinate analysis (PCoA) of bacterial ASV Bray–Curtis distances for different treatment. P, C, and B represent hydroxyapatite addition, no amendments, and biochar addition, respectively. L, N, and H represent low planting density, no planting, and high planting density, respectively.

In Figure 2, the first and second ordination axes explained 21% and 10.5% of the total variation, respectively. On the first ordination axis, the clusters of BH, BL, and BN in B treatments were similar, while the clusters of C treatments and P treatments were similar. On the second axis, the clustering of B treatments and C treatments was similar, but there was a clustering crossover between C and P treatments. Therefore, we conclude that there were substantial differences between the bacterial taxa present in the treatments P, C, and B, and the change of soil bacterial communities caused by biochar was higher than that caused by HAP, while the density of sweet sorghum had little effect.

### 3.3. Effects of SRAs and Plant Cultivation on Bacterial Community Composition

Histograms of relative abundances (RA) (Figure S5A) showed that Actinobacteria (36.42–50.17%) was the most abundant bacterial phylum in all samples, followed by Proteobacteria (26.51–31.63%), Acidobacteria (6.74–11.23%), Chloroflexi (6.22–9.81%), Gemmatimonadetes (2.78–5.45%), Bacteroidetes (1.62–2.10%), Patescibacteria (0.84–3.06%), Firmicutes (1.14–2.33%), Cyanobacteria (0.36–2.66%), and Nitrospirae (0.37–0.86%). These top ten phyla accounted for 98.11% of the total soil bacteria (Table S7). In order to further analyze the influence of different treatments on the RA of dominant phyla, the K–W test was conducted to test the difference in bacterial abundance. The results showed that there were significant differences in Actinobacteria (*p* = 0.009), Acidobacteria (*p* = 0.007), Gemmatimonadetes (*p* = 0.014), Patescibacteria (*p* = 0.016), and Nitrospirae (*p* = 0.037).

The RA of the top ten orders was calculated (Figure S6). The most abundant order was Rhizobiales (6.13–9.29%), followed by Gaiellales (5.02–7.11%), Betaproteobacteriales (4.33–8.76%), Micromonosporales (4.36–6.80%), Frankiales (4.13–6.81%), Micrococcales (3.86–6.16%), Myxococcales (3.12–4.94%), Gemmatimonadales (2.74–5.42%), Propionibacteriales (2.53–4.87%), Acidobacteriale (2.18–5.27%) (Table S8). There were significant differences in Rhizobiales (*p* = 0.018), Betaproteobacteriales (*p* = 0.028), Micrococcales (*p* = 0.017), Myxococcales (*p* = 0.048), Gemmatimonadales (*p* = 0.016), and Acidobacteriales (*p* = 0.022).

The RA of the top 15 genera was presented in Table S9. The most abundant genus was *Sphingomonas* (1.58–3.57%), followed by *Gemmatimonas* (1.67–2.83%), *Nocardioides* (1.27–2.53%), *Actinoplanes* (1.61–2.39%), *Streptomyces* (1.45–2.27%), *Mycobacterium* (1.32–2.70%), Subgroup_6 (0.89–3.36%), KD4-96 (1.37–2.40%), *Saccharimonadales* (0.71–2.55%), *Amycolatopsis* (0.34–3.42%), *Bradyrhizobium* (1.13–1.84%), 67-14 (0.83–2.11%), *Haliangium* (0.49–1.69%), *Bacillus* (0.75–1.42%), and *Burkholderia–Caballeronia–Paraburkholderia* (0.56–3.34%). There were significant differences in *Gemmatimonas* (*p* = 0.011), *Nocardioides* (*p* = 0.047), *Mycobacterium* (*p* = 0.005), Subgroup_6 (*p* = 0.042), *Saccharimonadales* (*p* = 0.012), *Amycolatopsis* (*p* = 0.008), *Bradyrhizobium* (*p* = 0.036), and *Haliangium* (*p* = 0.040).

At the phylum level, compared with N treatment, L and H treatment increased the RA of Actinobacteria, whereas they decreased the RA of Acidobacteria. In addition, B treatment increased the RA of Patescibacterias, but decreased the RA of Nitrospirae (Figure S5 and Table S7).

At the order level, cultivation of sweet sorghum increased the RA of Micrococcales, but decreased the RA of Acidobacteriales. In addition, B treatment increased the RA of Rhizobiales and Acidobacteriales and decreased the RA of Betaproteobacteria, while P treatment showed no significant effect on the dominant bacterial orders (Figure S6 and Table S8).

**Figure 3:**
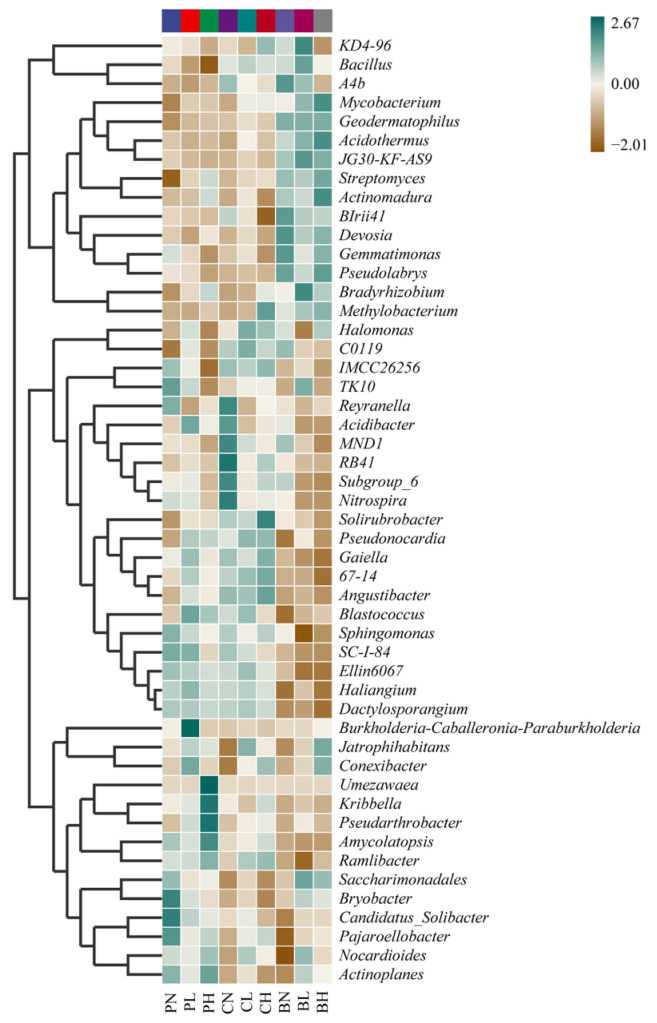
The heatmap showing the abundance of the top 50 genera. Color codes were based on standardization (row) of species abundance (see scale on the top right). P, C, and B represent hydroxyapatite addition, no amendments, and biochar addition, respectively. L, N, and H represent low planting density, no planting, and high planting density, respectively.

As shown in Figure 3, the composition of bacterial genera was similar between P and C treatments, while B treatment showed more significant differences. The RA of *Geodermatophilus*, *Acidothermus*, JG30-KF-AS9, *Streptomyces*, *Actinomadura*, BIrii41, *Devosia*, *Gemmatimonas*, *Pseudolabrys*, and *Bradyrhizobium* in B treatments (BL, BH, and BN) were higher than in P treatments (PL, PH, and PN) and C treatments (CL, CH, and CN). The RA of *Saccharimonadales*, *Bryobacter*, *Candidatus_Solibacter*, *Pajaroellobacter*, and *Actinoplanes* was increased by HAP in the soil without plant cultivation (PN), and the RA of *Umezawaea*, *Kribbella*, *Pseudarthrobacter*, and *Amycolatopsis* was increased by HAP in the soil with high-density cultivation (PH).

Sorghum planting density also affected the RA of some genera, enriching *Halomonas*, *Pseudonocardia*, and *Ramlibacter* in the soil without SRAs, but diminishing *Reyranella*, *Acidibacter*, MNDI, RB41, Subgroup_6, and *Nitrospira*.

### 3.4. MetagenomeSeq Analysis

As shown in Figure S7, we tried to identify the ASVs by differences among the sample groups and then determined whether these differences tended to be enriched at different classification levels. The differences were mainly found in the Acidobacteria, Actinobacteria, Chloroflexi, Gemmatimonadetes, Proteobacteria, Bacteroidetes, and Patescibacteria.

In the treatments with plant cultivation, the addition of biochar increased the ASVs of Acidobacteria, Actinobacteria, Chloroflexi, Gemmatimonadetes, and Proteobacteria. However, HAP enriched more ASVs in the soil without plant cultivation. Compared with C treatment (CH and CL), the number of ASVs upregulated by BH and BL was significantly higher than that by PH and PL, respectively, but the opposite tendency was observed in the N treatment (BN, PN, and CN) (Figure S7A).

The increase of planting density caused the enrichment of ASVs in B and C treatments, but was not significant in P treatment. In both C and B treatments, the number of ASVs upregulated by CL and BL was lower than that of CH and BH compared with CN and BN, respectively. However, the number of ASVs upregulated by PL and PH was not significantly different compared with PN (Figure S7B).

### 3.5. LEfSe Analysis

**Figure 4:**
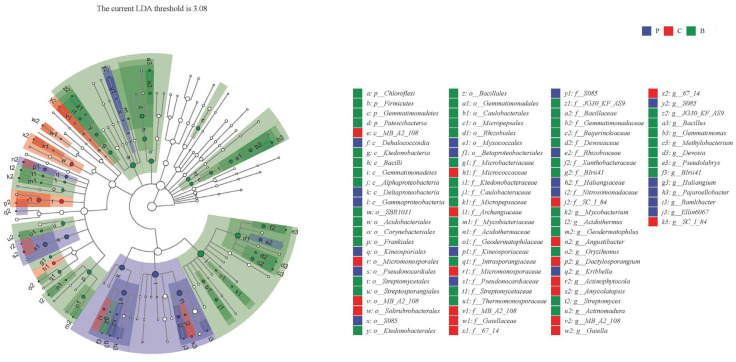
LEfSe taxonomic cladogram. The colored nodes from inner circle to outer circle represent the hierarchical relationship of all taxa from the phylum to the genus level. Taxa enriched in B group are shown in green while taxa enriched in C group are shown in red and taxa enriched in P group are colored in blue. Taxa with no significant difference between groups are hollow nodes, while these taxa with significant difference between groups are the nodes of other colors (bule, green and red). P, C, and B represent hydroxyapatite addition, no amendments, and biochar addition, respectively.

To identify the biomarkers of soil microbiota in different groups, we performed LEfSe analysis among P, C, and B treatments (Figure 4). LDA results showed 48 discriminative features in B treatment (LDA > 3.08, *p* < 0.05), and Ktedonobacterales, Saccharimonadia, Rhizobiales, Frankiales, and Corynebacteriales were the main taxa. P treatment showed 26 dominant taxa (LDA > 3.08, *p* < 0.05), and the major bacteria were Pseudonocardiales, Gammaproteobacterial, Betaproteobacteriales, Myxococcales, and Micrococcaceae. C treatment showed 22 dominant bacteria (LDA > 3.08, *p* < 0.05), and the major taxa were Deltaproteobacteria, Pyrinomonadales, and Solirubrobacterale (Figure S8). Then, an evolutionary clustering analysis diagram was delivered to identify major microflora by taxonomy (Figure 4). In the cladogram, Rhizobiales, Gemmatimonadetes, Saccharimonadia, Ktedonobacteria, Corynebacteriales, and Frankiales were in green parts, and Pyrinomonadales, Azospirillales, Kineosporiales, MB_A2_108, Rubrobacterales, and Solirubrobacterales were in the red area, Pseudonocardiales, Gammaproteobacteria, Haliangiaceae, Betaproteobacteriales were in blue area, which represented B, C, and P treatments, respectively. Overall, these results indicated that the marker species of soil bacteria were altered by HAP or biochar.

We performed LEfSe analysis among L, H, and N treatments. LDA results showed five discriminative features in L treatment (LDA > 3, *p* < 0.05), and *Nocardioides*, *Subdoligranulum*, *Cellulosimicrobium*, *Shinella*, and *Aquabacterium* were the main taxa. H treatment showed 12 dominant bacteria (LDA > 3, *p* < 0.05), and the major taxa were *Clostridia*, Clostridiales, *Sulfuriferula*, Sulfuricellaceae, *Flavonifractor*, *Bradyrhizobium*, Mycobacteriaceae, *Mycobacterium*, Nocardioidaceae, Micrococcales, Actinobacteria, and Actinobacteria. N treatment showed seven dominant bacteria (LDA > 3, *p* < 0.05), and the major taxa were *MND1*, Micavibrionales, Gemmatimonadetes, Gemmatimonadetes, Gemmatimonadales, Gemmatimonadaceae, and Acidobacteria (Figure S10). More details of the major taxa by taxonomy could be found in an evolutionary clustering analysis diagram (Figure S9).

### 3.6. Correlations between Soil Properties and Bacterial Abundance

**Figure 5:**
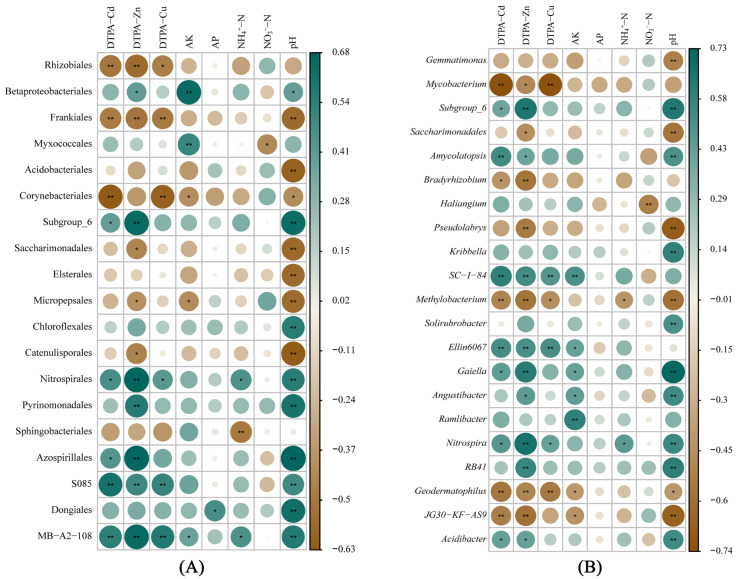
Correlation heatmaps between soil physicochemical properties and bacterial abundance (**A**) order; (**B**) genus. The *p* value filter is set to 0.05 and the *R* value filter is set to 0.5. The color and circle size indicate the *R* value, which is correlation, and the asterisk indicates significance.

Soil physicochemical properties and bacterial abundance data were used to generate correlation heatmaps (Figure 5A). At the order level, DTPA-Cd, -Zn, -Cu, and pH were negatively correlated with marker species (Rhizobiales, Frankiales, and Corynebacteriales) and positively correlated with S085, MB-A2-108, Azospirillales, and Pyrinomonadales. AK was positively correlated with Betaproteobacteriales, Myxococcales, and MB-A2-108, while negatively correlated with Corynebacteriales and Micropepsales. NH_4_^+^-N was positively correlated with Nitrospirales and MB-A2-108 and negatively correlated with Sphingobacteriales. NO_3_^−^-N was negatively correlated with Myxococcales.

Soil bacteria at the genus level showed similar correlations with soil properties (Figure 5B). *Gemmatimonas*, *Geodermatophilus*, JG30-KF-AS9, *Saccharimonadales*, *Methylobacterium*, *Pseudolabrys*, and *Bradyrhizobium* were negatively correlated with pH and DTPA-Cd, -Cu, and -Zn. NH_4_^+^-N was positively correlated with *Nitrospira* and negatively correlated with *Methylobacterium*. NO_3_^−^-N was negatively correlated with *Haliangium*.

## 4. Discussion

### 4.1. Effects of Biochar and HAP on Bacterial Communities by Altering Soil Properties

Previous studies have shown that the SRAs biochar and HAP can act both directly on soil microorganisms (e.g., provide nutrients and habitats) and indirectly via changing soil physicochemical properties (particularly charosphere) and plant growth [8,30,31,40,41,42]. Soil bacterial communities vary with soil properties [43], especially pH [44,45]. For example, many studies supposed that *Gemmatimonas* [46], *Saccharimonadales* [47], and *Bradyrhizobium* [48] were negatively correlated with soil pH. Additionally, the fixation of heavy metals by SRAs could affect soil bacterial communities by alleviating the toxicity of heavy metals and improving soil quality [49]. Contrary to previous studies that have found increased diversity and richness of soil bacterial communities by biochar [17,50,51] and HAP [8], our present study found negative effects of biochar and HAP on soil community diversity and richness. HAP increased soil DTPA-Cd, which was negatively correlated with soil bacterial community diversity. Previous studies have shown that the immobilization of soil Cd by HAP is dependent on the dose of HAP [34,52]. Here, we confirm that the dose of 0.1% is too low to immobilize Cd under field conditions. Additionally, HAP can increase soil pH and available P [7], but we did not observe similar results. Particularly, HAP contains 0.5 mg kg^−1^ Cd, which may release into the soil during plant growth. Although biochar generally increases the pH of acidic soil, the effects vary with the type and dose of biochar [53]. Our results found that, in some cases, biochar showed decreasing effects on soil pH and AK. The adsorption of biochar may be the reason for the decrease of soil available K. Biochar may contain toxic substances such as PAHs and toxic metals [54,55,56,57], which may damage soil microorganisms.

Biochar and HAP may change the abundance of specific bacteria. We found that soil properties were associated with the abundance of certain bacteria enriched in the soil with SRAs. For example, the RA of *Gemmatimonas*, *Geodermatophilus*, *Saccharimonadales*, *Methylobacterium*, *Pseudolabrys*, and *Bradyrhizobium*, JG30_KF_AS9, which were marker species in the soils amended with biochar, was negatively correlated with available metals (DTPA-Cd, -Zn, and -Cu) and pH in the soil. Similarly, the RA of SC_I_84, *Amycolatopsis*, and Ellin6067, which were marker species in the soil amended with HAP, was positively correlated with pH and soil available metals (DTPA-Cd, -Zn, and -Cu). Moreover, PCoA-envfit analysis also showed DTPA-Cd, -Cu, and -Zn and AK had great influences on the separation of B, P, and C treatments. Therefore, we conclude that biochar and HAP can drive the selection of some specific bacteria via altering soil properties.

### 4.2. Effects of Planting Density on Soil Bacterial Communities

Crops not only directly influence the community structure of symbiotic microorganisms, but also indirectly drive the succession of soil microorganisms by altering the properties of the growing soil, particularly the rhizosphere soil via secreting root exudates. In our study, plant cultivation altered soil bacterial composition, reflected by the changes in the RA of some specific taxa. For example, we found increased RA of beneficial bacteria *Bradyrhizobium* and some soil bacteria involved in the decomposition of organic carbon, such as Actinobacteria (*Mycobacterium*, Nocardioidaceae, and Micrococcales) and *Clostridia* [58]. A previous study found that fallow soil had higher carbon content, greater microbial biomass, and higher microbial diversity compared to the cultivated soil [59]. Our findings confirm that plant cultivation helps to drive the succession of soil bacteria during the phytoremediation program. However, the planting density of sorghum did not affect the diversity and richness of soil bacterial community (Table S1), and even decreased the Faith_pd index in B and C treatments (Table S6). Overall, planting sweet sorghum had weaker effects on soil bacterial community diversity and richness than SRAs. One possible reason may be that SRAs caused more significant changes in soil properties, which generally impact soil bacterial community diversity more strongly than plants [60].

Additionally, sorghum cultivation promoted the upregulation of soil ASVs (classified as Acidobacteria, Actinobacteria, Chloroflexi, and Proteobacteria) in the soil amended with biochar, but had a small effect in the soil amended with HAP (Figure S7). Biochar can serve as a refuge for fungi and bacteria. Pore sizes of biochar could satisfy the space for soil microorganisms to enter and protect microorganisms from external factors such as grazing predators, desiccation, adverse pH, or toxic substances in soil [61,62]. In contrast, HAP cannot provide a good place for bacterial colonization. In our study, sorghum cultivation did not affect ASVs upregulation in the soil with HAP addition. However, the effects of HAP on ASVs were significantly greater than that of biochar in the soil without sorghum cultivation (Figure S7), indicating that HAP has a great direct effect on soil bacterial ASVs, while plant cultivation had no significant indirect effect.

### 4.3. Effects of SRAs and Plant Cultivation on Specific Bacteria

In our study, biochar caused the enrichment of some dominant taxa (Figure 3 and Figure S8). Possible reasons can be ascribed to decreased Cd mobility, improved soil properties (nutrients and carbon source), and enhanced plant growth by biochar. These bacteria are reported to promote plant growth and increase plant tolerance to toxic metals. Several previous studies found that *Bacillus* sp. facilitated the immobilization of Cd [63], *Bacillus subtilis* C (225)(MK334652) reduced the bioavailability of heavy metals (Zn, Cr, and Cu) [64], whereas *Bacillus thuringiensis* decreased the phytoavailability of soil Pb [65]. *Bradyrhizobium japonicum* was able to enhance the growth of lettuce seedlings under heavy metal stress [66]. *Streptomyces pactum* decreased the antioxidant activities and lipid peroxidation in wheat and mitigated metal stress in contaminated soils [67]. Siderophores produced by *Streptomyces* sp. played a significant role in tolerance to Cd^2+^ [68]. *Methylobacterium* alleviated heavy metal stress induced in plants [69]. *Geodermatophilus* species have been reported to occur in heavy metal-contaminated soils [70,71], which may have a potential for soil bioremediation. Similar to our results, the members belonging to JG30-KF-AS9, *Acidothermus*, Subgroup_6, KD4-96, and *Bradyrhizobium* were also observed to dominate in the soil contaminated with Cr, Ni, and Cu [72]. In conclusion, biochar can enrich some beneficial bacteria involved in heavy metal resistance and plant-growth promotion, thus reducing heavy metal toxicity and promoting plant growth, which may represent a mechanism for biochar-amendment effects.

Similarly, the addition of HAP also enriched some important bacteria, such as *Candidatus_Solibacter* and *Amycolatopsis* (Figure 3). *Candidatus_Solibacter* has been shown to comprise the core microbiome of various soils polluted with different toxic metals [73,74,75,76]. A previous study showed that *Amycolatopsis* exhibited possible tolerance to both acid soils and the presence of certain metals (As, Cr, and Ni) [8]. Other genera, such as Actinoplanes [77], *Pajaroellobacter* [78], and *Pseudarthrobacter* [79], were also observed to occur in heavy metal-contaminated soils. The enrichment of these microorganisms by HAP indicates their potential use in soil remediation jointly with HAP.

In addition, plant cultivation enriched some specific taxa, *Halomonas*, *Pseudonocardia*, and *Ramlibacter*. Some members of *Halomonas* belong to plant growth-promoting bacteria, with significant tolerance to salt and heavy metals [80]. *Pseudonocardia* and *Ramlibacter* show a high tolerance to heavy metal contamination [81,82]. These taxa may contribute to the growth of sweet sorghum in Cd-contaminated soil.

### 4.4. Potential Functions of Marker Species in Soil C and P Cycling

The enriched marker species by biochar may participate in C cycling, particularly in the utilization of the carbon source in biochar. Previous studies have found that *JG30-KF-AS9* [83], Ktedonobacteraceae [84], Bacillales [85], *Gemmatimonas* [86], *Acidothermus* (Frankiales) [87], *Actinomadura* [88], Micropepsaceae [89], and *Oryzihumus* [90], the marker species in B treatment, are associated with the mineralization of soil organic carbon. *Actinomadura* [88], *Acidothermus* [87], and Ktedonobacteria [84] can decompose cellulose. These bacteria may enhance the degradation of biochar and thus negatively affect the immobilization of toxic metals by biochar.

HAP-enriched marker species may contribute to soil microbial P turnover and phosphate solubility, such as *Candidatus_Solibacter* [91], *Amycolatopsis* [92], and Myxococcales [93] (order). Some members of Saccharimonadales are recognized as phosphorus-solubilizing bacteria (PSB) [94]. Similar to our present results, previous studies found Micrococcaceae (family) and Myxococcales (order) were the marker species in the soil amended with HAP [31,95]. These enriched species may modify the immobilization of heavy metals by HAP and the absorption of P by plants, which need to be evaluated in future work. Finally, the exact functions of these marker species in C and P cycling must be confirmed by various experimental techniques.

## 5. Conclusions

Based on our current experiment, several conclusions can be drawn. First, biochar and HAP substantially altered the assembly of the soil bacterial community, evidenced by the decrease in the α diversity and the changes in community composition. Comparatively, planting with sweet sorghum had a negligible influence on soil bacterial diversity, but caused some alterations in the abundance of some specific taxa. The changes in soil properties induced by biochar and HAP may partly explain their effects on soil bacteria, particularly the taxa sensitive to soil condition changes. The enriched bacteria may have specific ecological functions in regulating plants’ growth and tolerance to Cd and nutrient cycling, which deserves more detailed investigations. Finally, our findings are based on a one-growing-season experiment, and long-term evaluation of soil microbial succession during soil remediation should be considered.

## Figures and Tables

**Table 1:** Physicochemical properties of the soil in the field.

pH	NH_4_^+^-N	NO_3_^−^-N	SOC	Available K	Available P	Total Cd	Total Zn
	mg kg^−1^	mg kg^−1^	g kg^−1^	mg kg^−1^	mg kg^−1^	mg kg^−1^	mg kg^−1^
5.04 ± 0.02	0.43 ± 0.19	3.84 ± 0.20	16.15 ± 0.63	130.83 ± 6.08	48.32 ± 15.28	0.47 ± 0.06	32.34 ± 7.34

Note: SOC, soil organic carbon.

## Data Availability

Not applicable.

## Supplementary Materials

The following supporting information can be downloaded at: https://www.mdpi.com/article/10.3390/toxics11010053/s1, Figure S1: Scanning electron microscopy of hydroxyapatite; Figure S2: Sequence length distribution diagram; Figure S3: The number of ASVs in different samples; Figure S4: The α diversity metrics showing Chao1, Observed_species, Shannon, and Simpson indexes; Figure S5: The relative abundance of the top 10 phyla as affected by different treatments; Figure S6: The relative abundance of the top 10 orders as affected by different treatments; Figure S7: Manhattan plot of metagenomeSeq analysis; Figure S8: LDA scores for soil bacterial communities; Figure S9: LEfSe taxonomic cladogram; Figure S10: LDA scores for soil bacterial communities. Table S1: Significance levels (*F* value) of sweet sorghum density, remediation agent type, and their interactions for measured variables according to a two-way ANOVA analysis; Table S2: Effects of different treatments on soil properties and Cd content in plants; Table S3: Pearson test for edaphic factors and cadmium content in plants; Table S4: Pearson test for edaphic factors and alpha diversity index; Table S5: Statistical table of sample sequencing amount; Table S6: Alpha diversity index; Table S7: The relative abundance (%) of the top 10 phyla as affected by different treatments; Table S8: The relative abundance (%) of the top 10 orders as affected by different treatments. Table S9: The relative abundance (%) of the top 15 genus as affected by different treatments.
