# Supplementary material for: Remediation Agents Drive Bacterial Community in a Cd-Contaminated Soil
Source: Toxics. 2023 Jan 4;11(1):53. doi: 10.3390/toxics11010053 (PMC9861843; doi:10.3390/toxics11010053)
Supplement: Supplementary file 1 [file toxics-11-00053-s001.zip › toxics-2151606-supplementary.pdf]

Table S1. Significance levels (*F* value) of sweet sorghum density, remediation agent type, and their interactions for measured variables according to a two-way ANOVA analysis

Variables	Sweet sorghum density (D)	SRA type (T)	D * T
Chao1	0.396	4.694**	2.346*
Faith_pd	3.583**	9.783***	3.282**
Goods_coverage	0.514	4.963**	2.675*
Observed_species	0.656	6.795**	2.282
Pielou_e	2.202	4.278**	0.434
Shannon	1.853	3.234*	0.337
Simpson	1.422	2.432	0.447
AK	7.401***	16.681***	2.881*
AP	0.570	0.547	0.347
DTPA-Cd	0.326	6.970***	0.167
TCLP-Cd	5.743**	0.726	1.442
DTPA-Zn	2.821*	2.473	1.357
NH_4^+-N	1.480	2.417	0.208
NO_3^--N	2.926*	2.796*	1.556
DTPA-Cu	1.116	4.968**	1.183
pH	1.737	2.985*	2.011
Cd (root)	0.401	2.520	0.455
Cd (stem)	0.000	2.043	0.437

Note: Asterisks indicate values that significantly differ in these treatments ($^{*}p < 0.1$, $^{**}p < 0.05$, $^{***}p < 0.01$).

Table S2 Effects of different treatments on soil properties and Cd content in plants

	pH	AK	AP	DTPA-Zn	NH_4^+-N	NO_3^--N	DTPA-Cu	TCLP-Cd	DTPA-Cd	Cd (root)	Cd (stem)	Cd (leaf)
PL	6.05±0.22ABa	118.9±8.56ABa	43.73±32.46Aa	3.50±1.29Bab	0.57±0.17ABab	3.03±1.02BCabc	2.04±0.36Aab	0.285±0.0553Cb	0.18±0.07ABa	1.04±0.62Aa	0.75±0.91Aa	—
PH	6.01±0.07ABa	123.18±3.35ABa	71.86±38.95Aa	4.53±1.82ABab	0.67±0.31Aa	2.37±0.27BCbc	2.07±0.06Aab	0.297±0.0682BCb	0.22±0.14Aa	1.60±1.24ABa	1.11±1.04Aa	—
PN	5.92±0.10ABab	123.92±10.60ABa	55.53±30.12Aa	4.46±1.76ABab	0.55±0.26ABab	2.39±0.91BCbc	2.14±0.15Aa	0.467±0.0636Aa	0.23±0.13Aa			
CL	5.87±0.22ABab	130.72±8.48Aa	44.70±27.97Aa	2.65±0.74Bb	0.42±0.07ABab	3.25±1.45ABCabc	1.82±0.31ABabc	0.297±0.1077BCb	0.09±0.006Ba	0.58±0.20Ba	0.62±0.24Aa	0.09±0.13
CH	5.98±0.23ABa	116.53±9.59Bab	47.33±14.95Aa	3.36±0.56Bab	0.50±0.17ABab	1.65±0.57Cc	1.89±0.09ABabc	0.294±0.0241BCb	0.09±0.008Ba	0.48±0.16Ba	0.35±0.06Aa	—
CN	6.12±0.15Aa	130.83±6.08Aa	52.94±15.28Aa	6.45±2.20Aa	0.43±0.19ABab	3.87±0.20ABab	1.93±1.05ABabc	0.397v0.0591ABab	0.10±0.02Ba			
BL	5.81±0.11BCab	103.53±4.04Cbc	44.07±0.68Aa	1.89±0.33Bb	0.30±0.12Bb	3.07±1.87BCabc	1.6±0.03Bbc	0.370±0.0682ABCab	0.08±0.005Ba	0.78±0.13ABa	0.28±0.28Aa	—
BH	5.59±0.06Cb	94.51±8.37Cc	46.84±4.43Aa	3.45±3.13Bab	0.55±0.09ABab	3.38±1.45ABabc	2.01±0.51Aabc	0.361±0.0773ABCab	0.11±0.08ABa	0.87±0.50ABa	0.20±0.34Aa	—
BN	6.02±0.32ABa	120.98±6.41ABa	48.37±2.02Aa	2.64±0.93Bb	0.41±0.14ABab	4.82±0.64Aa	1.55±0.02Bc	0.376±0.0721ABCab	0.08±0.02Ba			

Note: The data in the figure are presented in the form of mean ± standard deviation, in mg/kg. Duncan's test results are listed in the table in alphabetical notation. Uppercase letters represent subset for $p < 0.1$ and lowercase letters represent subset for $p < 0.05$. P, C, and B represent hydroxyapatite addition, no amendments, and biochar addition, respectively. L, N, and H represent low planting density, no planting, and high planting density, respectively.

Table S3. Pearson test for edaphic factors and cadmium content in plants

	AK	AP	DTPA-Cd	DTPA-Zn	NH_4^+-N	NO_3^--N	DTPA-Cu	pH	Cd(root)	Cd(stem)	Cd(leaf)
AK	1	0.192	-0.014	0.108	0.129	-0.008	-0.03	**0.429****	-0.112	0.306	0.168
AP		1	-0.145	-0.022	0.031	0.116	**0.352***	0.045	-0.049	-0.136	0.259
DTPA-Cd			1	**0.522*****	**0.462****	-0.118	**0.619*****	0.186	**0.925*****	**0.724*****	-0.109
DTPA-Zn				1	0.151	0.142	**0.541*****	**0.338***	**0.673*****	0.259	0.039
NH_4^+-N					1	-0.028	**0.366***	0.152	0.179	**0.434***	-0.041
NO_3^--N						1	0.071	-0.037	0.232	0.182	0.343
DTPA-Cu							1	0.01	**0.510****	0.287	0.147
pH								1	0.207	0.377	0.276
Cd(root)									1	**0.651*****	-0.032
Cd(stem)										1	0.143
Cd(leaf)											1

Note: Asterisks indicate values that significantly differ in these treatments (* $p < 0.1$, ** $p < 0.05$, *** $p < 0.01$).

Table S4. Pearson test for edaphic factors and alpha diversity index

	AK	AP	DTPA-Cd	DTPA-Zn	NH_4^+-N	NO_3^--N	DTPA-Cu	pH
Chao1	0.513***	0.465**	-0.085	0.139	-0.043	-0.061	0.154	0.424**
Faith_pd	0.585***	0.194	-0.340*	0.228	-0.027	0.227	-0.134	0.632***
Goods_coverage	-0.459**	-0.474**	-0.016	-0.076	0.036	0.115	-0.228	-0.262
Pielou_e	0.043	0.179	-0.686***	-0.161	-0.225	0.208	-0.308	0.092
Observed_species	0.592***	0.381*	-0.212	0.210	0.001	0.039	0.034	0.628***
Shannon	0.301	0.307	-0.616***	-0.033	-0.172	0.172	-0.221	0.354*
Simpson	0.008	0.203	-0.684***	-0.253	-0.216	0.139	-0.283	-0.087

Note: Asterisks indicate values that significantly differ in these treatments ($^{*}p < 0.1$, $^{**}p < 0.05$, $^{***}p < 0.01$).

Table S5. Statistical table of sample sequencing amount

SampleID	Input	Filtered	Denoised	Merged	Nonchimeric	Nonsingleton
PL1	74847	70177	64713	47720	40574	38318
PL2	68246	64138	59432	45186	38737	36904
PL3	78864	73878	69063	52603	45077	43465
PH1	79121	74453	70275	58095	50014	48574
PH2	81521	75726	70818	54766	45734	43932
PH3	89048	83206	78009	61417	52074	50201
PN1	87080	82197	78722	64821	58928	57565
PN2	87639	82479	78096	62832	55459	53908
PN3	76014	71364	66419	49615	43104	41218
CL1	72453	67212	63046	50454	44790	43248
CL2	78369	73435	68640	52110	43580	41746
CL3	75613	70479	65398	50779	44918	42613
CH1	70441	65732	61111	45901	38580	36698
CH2	65852	61603	56675	42577	36413	34285
CH3	69932	65784	60921	45685	38775	36681
CN1	75721	71144	65716	47939	41090	38839
CN2	75918	71407	65608	48136	41467	38545
CN3	73374	68754	63076	45476	39004	36327
BL1	75495	69567	65435	51372	45311	43901
BL2	85314	79516	74336	59033	52496	50989
BL3	78156	73350	69019	56347	50539	49230
BH1	81399	76209	71690	53133	45436	43614
BH2	66041	62290	57908	43421	37237	35755

BH3	72937	68519	64475	51918	45873	44614
BN1	64866	60687	55692	38767	32838	31081
BN2	68467	64433	59464	43010	37348	35295
BN3	71259	67290	62231	46044	40128	38091

Table S6. Alpha diversity index

Sample	Chao1	Faith_pd	Goods_coverage	Observed_species	Pielou_e	Shannon	Simpson
PL	4494.37±292.87ABCab	257.41±12.77Bb	0.97±0.0031A-Dabc	3833.13±218.00Bbc	0.89±0.015ABab	10.58±0.25Bab	0.998±0.0014ABab
PH	4796.90±316.62Aa	246.88±10.20Bbc	0.96±0.0036Dc	3971.03±188.92ABab	0.87±0.024Bc	10.45±0.35Bb	0.996±0.0032Bb
PN	4534.68±301.11ABCab	240.97±14.86BCbc	0.97±0.0038BCDabc	3733.17±221.22BCbc	0.89±0.004Aab	10.57±0.06Bab	0.998±0.0005Aab
CL	4628.76±420.60ABab	255.24±16.88Bb	0.96±0.0047CDbc	3891.43±327.86Babc	0.90±0.007Aa	10.69±0.18ABab	0.999±0.0002Aab
CH	4349.99±89.11BCab	256.84±8.48Bb	0.97±0.0011ABCab	3851.57±124.23Bbc	0.89±0.011Aab	10.63±0.17ABab	0.998±0.0006Aab
CN	4835.27±52.57Aa	280.73±2.15Aa	0.96±0.0013Dbc	4221.13±96.47Aa	0.90±0.004Aa	10.89±0.08Aa	0.999±0.0001Aa
BL	4491.90±225.62ABCab	243.38±11.34BCbc	0.97±0.0025A-Dabc	3724.47±172.68BCbc	0.90±0.002Aa	10.63±0.08ABab	0.999±0.0001Aab
BH	4143.52±167.13Cb	228.59±8.19Cc	0.97±0.0017ABab	3509.10±126.86Cc	0.89±0.004Aa	10.54±0.09Bb	0.998±0.0003Aab
BN	4170.66±315.70Cb	252.76±8.77Bb	0.97±0.0039Aa	3696.67±217.33BCbc	0.90±0.004Aa	10.64±0.12ABab	0.999±0.0001Aab

Duncan's test results are listed in the table in alphabetical notation. Uppercase letters represent subset for $p < 0.1$ and lowercase letters represent subset for $p < 0.05$. P, C, and B represent hydroxyapatite addition, no amendments, and biochar addition, respectively. L, N, and H represent low planting density, no planting, and high planting density, respectively.

Table S7. The relative abundance (%) of the top 10 phyla as affected by different treatments.

	PL	PH	PN	CL	CH	CN	BL	BH	BN
Actinobacteria	42.06±3.67	50.17±4.75	38.26±0.9	42.94±4.11	43.85±2.91	38.2±2.14	40.47±1.61	42.19±0.8	36.72±1.3
Proteobacteria	31.63±5.51	26.51±3.29	29.36±1.86	28.38±1.81	30.54±6.03	29.9±1.93	27.55±0.24	28.39±1.48	29.72±0.68
Acidobacteria	8.2±1.08	6.74±0.23	10.89±0.53	7.66±0.84	7.74±0.91	11.23±2.11	8.42±0.18	7.94±0.39	10.65±1.11
Chloroflexi	6.92±0.81	6.22±0.64	6.88±2.05	7.08±1.66	7.01±1.63	7.37±1.47	9.81±0.7	7.4±0.69	9.02±1.9
Gemmatimonadetes	3.52±0.42	2.78±0.4	3.9±0.86	3.63±0.41	3.53±0.33	4.43±0.34	3.8±0.46	4.32±0.31	5.45±0.1
Bacteroidetes	1.79±0.35	2.01±0.25	1.99±0.56	1.94±0.15	1.62±0.2	1.88±0.21	2.1±0.05	2.07±0.03	1.76±0.21
Patescibacteria	1.62±0.92	1.78±0.21	2.6±1.32	1.56±0.14	0.95±0.27	0.84±0.23	3.06±0.56	2.62±0.78	1.52±0.2
Firmicutes	1.5±0.41	1.14±0.26	1.57±0.41	1.93±0.48	1.76±0.35	1.7±0.46	2.21±0.4	2.33±0.8	1.72±0.22
Cyanobacteria	0.39±0.19	0.96±1.27	2.16±1.3	2.66±3.07	0.36±0.11	1.05±0.69	0.59±0.23	0.37±0.1	0.64±0.46
Nitrospirae	0.58±0.14	0.45±0.06	0.6±0.07	0.52±0.17	0.56±0.17	0.86±0.34	0.38±0.03	0.37±0.02	0.53±0.12
Others	1.8±0.48	1.25±0.27	1.8±1.25	1.69±0.24	2.09±0.32	2.56±0.5	1.6±0.08	2±0.45	2.26±0.61

Note: The data in the figure are presented in the form of mean ± standard deviation. The K-W test results are described in the text. P, C, and B represent hydroxyapatite addition, no amendments, and biochar addition, respectively. L, N, and H represent low planting density, no planting, and high planting density, respectively.

Table S8. The relative abundance (%) of the top 10 orders as affected by different treatments.

	PL	PH	PN	CL	CH	CN	BL	BH	BN
Rhizobiales	6.62±0.39	7.06±0.86	6.13±0.6	6.52±0.12	9.25±3.68	6.75±0.77	9.63±1.13	9.67±0.79	9.69±0.77
Gaiellales	7.11±0.5	5.8±0.3	6.53±1.27	6.14±0.74	5.7±0.23	5.02±0.23	5.48±0.59	6.46±0.4	5.64±0.78
Betaproteobacteriales	8.76±5.43	5.24±0.38	5.77±0.74	6.21±0.4	6.3±0.21	6.26±0.52	4.33±0.68	4.43±0.9	4.55±0.49
Micromonosporales	5.45±0.71	6.8±1.98	5.41±0.48	5.88±1.03	5.38±0.6	5.66±0.62	4.85±0.5	4.36±1.03	4.72±1.34
Frankiales	4.91±1.1	4.89±0.8	4.22±0.53	5.6±1.5	4.75±0.31	4.13±0.93	6.06±0.65	6.81±0.26	5.14±0.45
Micrococcales	4.75±0.73	5.93±1.08	3.86±0.27	4.61±0.52	6.02±0.47	4.4±0.6	5.5±0.53	6.16±0.75	4.84±0.4
Myxococcales	4.62±0.79	4.25±0.51	4.57±0.31	4.94±1	3.99±0.34	4.38±0.13	3.87±0.7	3.25±0.39	3.12±0.55
Gemmatimonadales	3.47±0.43	2.74±0.38	3.87±0.85	3.59±0.42	3.47±0.26	4.34±0.33	3.77±0.45	4.29±0.31	5.42±0.1
Propionibacteriales	3.87±0.35	4.87±0.55	3.69±0.9	4.08±0.65	4.06±0.61	3.58±0.24	4.08±0.77	3.39±0.34	2.53±0.21
Acidobacteriales	3.02±1.5	2.74±0.44	5.27±0.88	2.77±0.64	2.18±0.99	2.88±0.83	4.19±0.18	4.52±0.13	5.23±1.59

Note: The data in the figure are presented in the form of mean ± standard deviation. The K-W test results are described in the text. P, C, and B represent hydroxyapatite addition, no amendments, and biochar addition, respectively. L, N, and H represent low planting density, no planting, and high planting density, respectively.

Table S9. The relative abundance (%) of the top 15 genus as affected by different treatments.

	PL	PH	PN	CL	CH	CN	BL	BH	BN
Sphingomonas	3.12±0.11	2.8±1.08	3.57±1.53	2.84±1.19	3.22±2.06	3.25±0.66	1.58±0.2	2.05±0.47	2.85±0.69
Gemmatimonas	2.01±0.24	1.67±0.36	2.29±0.34	2.06±0.19	1.67±0.33	1.92±0.32	2.23±0.13	2.63±0.16	2.83±0.09
Nocardioides	2.17±0.34	2.47±0.41	2.28±0.61	2.41±0.46	2.07±0.12	1.68±0.35	2.53±0.38	1.97±0.25	1.27±0.21
Actinoplanes	2.02±0.82	2.39±0.92	2.3±0.52	1.9±0.73	1.65±0.12	1.71±0.27	2.14±0.47	1.96±0.84	1.61±0.46
Streptomyces	1.81±0.13	2.03±0.61	1.45±0.13	1.82±0.42	1.84±0.49	1.72±0.15	2.09±0.28	2.27±0.17	2.16±0.05
Mycobacterium	1.7±0.33	1.68±0.08	1.32±0.05	1.95±0.17	1.96±0.16	1.52±0.28	2.36±0.28	2.7±0.19	1.92±0.16
Subgroup_6	2±1.02	1.33±0.1	1.78±0.78	1.74±0.89	2.3±0.99	3.36±1.43	1.02±0.24	0.89±0.11	2.32±0.7
KD4-96	1.67±0.51	1.47±0.44	1.72±0.66	1.51±0.64	2.09±0.5	1.64±0.2	2.4±0.05	1.37±0.06	1.89±0.19
Saccharimonadales	1.43±0.79	1.61±0.19	2.23±1.11	1.31±0.13	0.72±0.23	0.71±0.24	2.55±0.31	2.23±0.77	1.19±0.04
Amycolatopsis	1.81±0.22	3.42±1.01	2.31±1.68	1.37±0.66	1.9±0.43	1.14±0.1	0.34±0.08	0.4±0.15	0.56±0.48
Bradyrhizobium	1.33±0.08	1.51±0.03	1.13±0.3	1.22±0.1	1.44±0.19	1.18±0.27	1.84±0.13	1.56±0.09	1.39±0.36
67-14	1.53±0.3	1.34±0.35	1.28±0.24	1.6±0.31	1.69±0.44	1.51±0.03	1.14±0.12	0.97±0.15	1.16±0.24
Haliangium	1.69±0.23	1.39±0.07	1.48±0.24	1.48±0.29	1.34±0.24	1.42±0.19	0.94±0.55	0.51±0.24	0.49±0.15
Bacillus	0.91±0.29	0.75±0.18	1.05±0.46	1.23±0.51	1.19±0.37	1.17±0.29	1.42±0.39	1.12±0.08	1.2±0.16
BurkholderiaCaballeroniaParaburkholderia	3.34±4.95	0.62±0.1	1±0.32	0.71±0.45	0.56±0.38	0.58±0.38	0.75±0.32	1.02±0.48	0.77±0.13
Others	71.46±2.55	73.51±3.06	72.8±1.15	74.84±3.07	74.35±0.18	75.49±1.2	74.68±0.67	76.36±1.11	76.39±0.58

Note: The data in the figure are presented in the form of mean ± standard deviation. The K-W test results are described in the text. P, C, and B represent hydroxyapatite addition, no amendments, and biochar addition, respectively. L, N, and H represent low planting density, no planting, and high planting density, respectively.

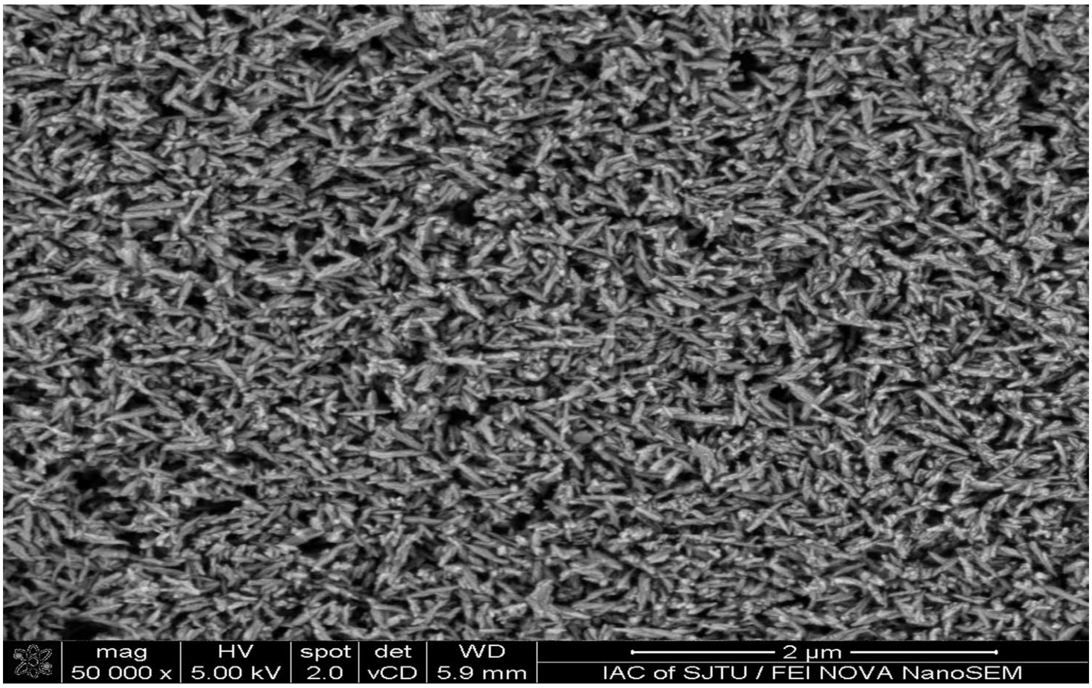

Figure S1. Scanning electron microscopy of hydroxyapatite

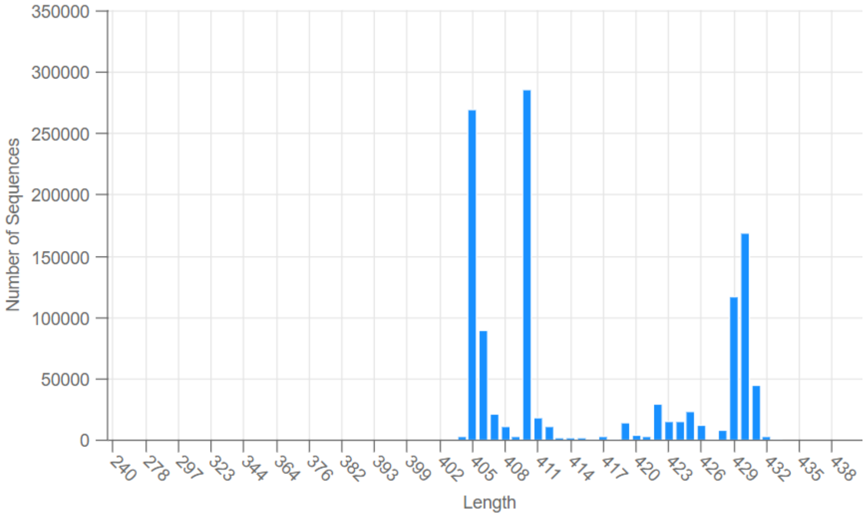

Figure S2. Sequence length distribution diagram. The abscissa is the length of the sequence and the ordinate is the number of the sequence.

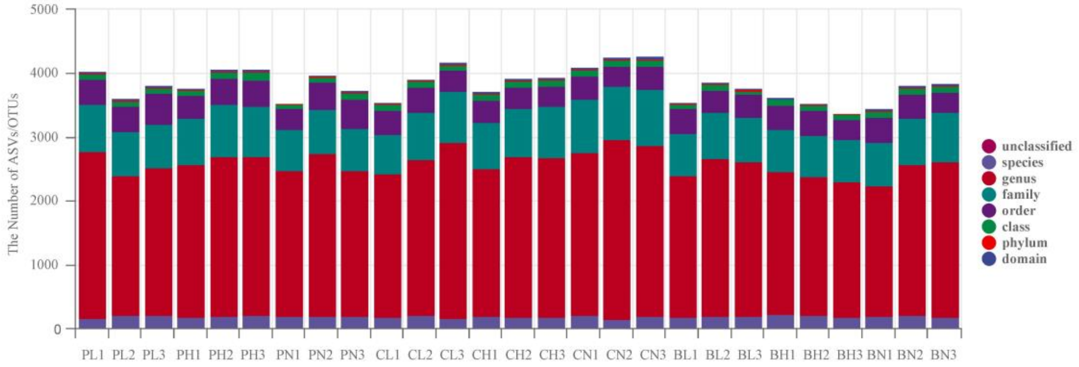

Figure S3. The number of ASVs in different samples. The abscissa represents the number of ASVs of each sample, and the ordinate represents the number of ASVs of domain, phylum, class, order, family, genus and species (the number of ASVs that can only be annotated to these levels; Default statistics up to species). Different classification levels are identified by different colors, and column heights correspond to the number of ASVs. This figure can intuitively display the annotation accuracy of each sample and the annotation situation of different samples. P, C, and B represent hydroxyapatite addition, no amendments, and biochar addition, respectively. L, N, and H represent low planting density, no planting, and high planting density, respectively.

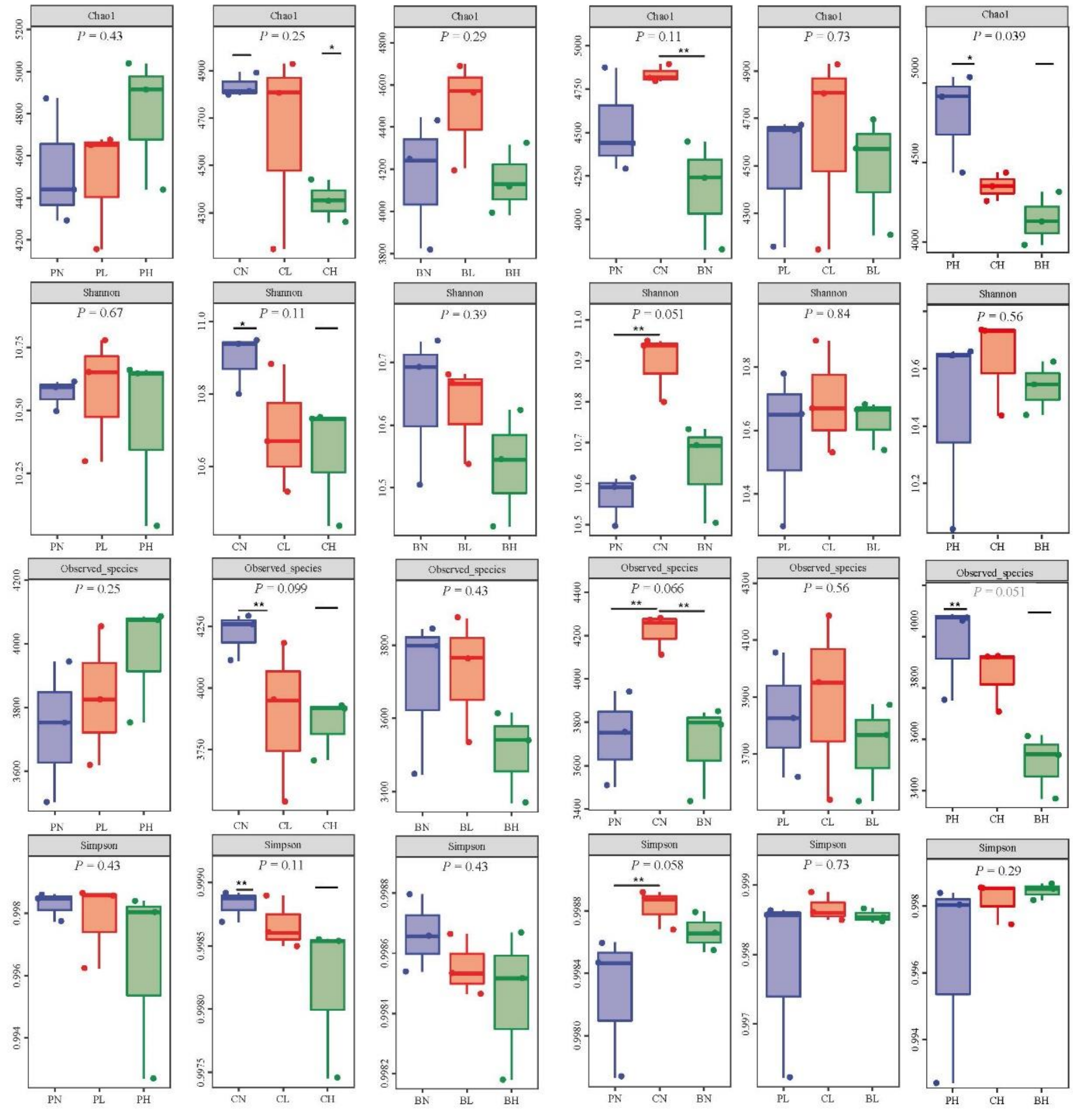

Figure S4. The α diversity metrics showing Chao1, Observed_species, Shannon, and Simpson indexes. The figure shows the *P*-values of K-W tests and marks the results of multiple tests (**P* < 0.1, ***P* < 0.05). P, C, and B represent hydroxyapatite addition, no amendments, and biochar addition, respectively. L, N, and H represent low planting density, no planting, and high planting density, respectively.

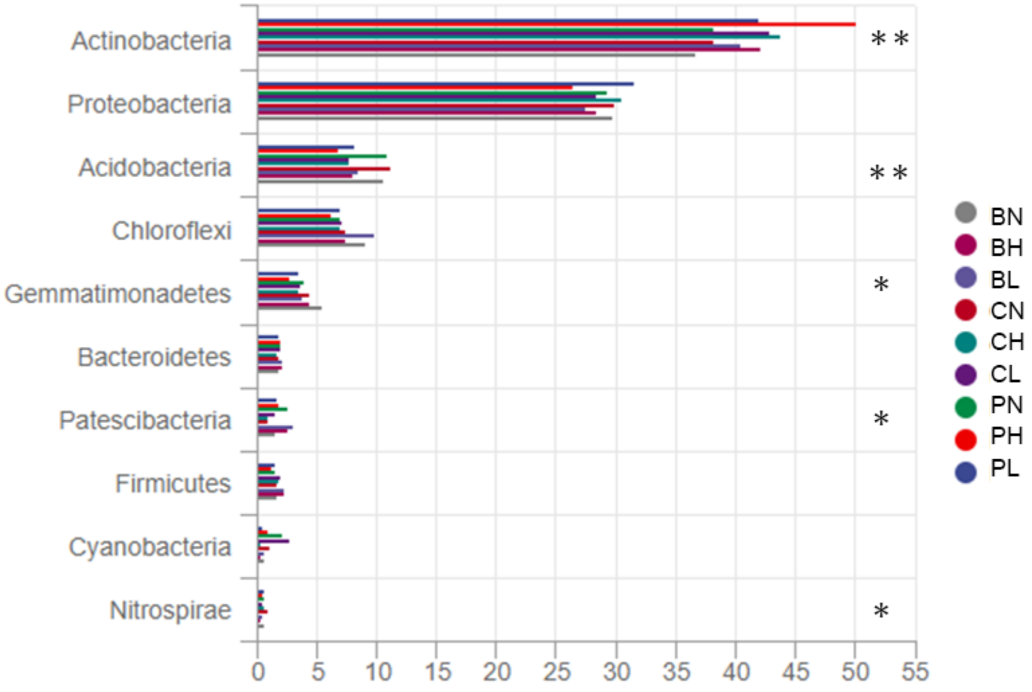

(A)

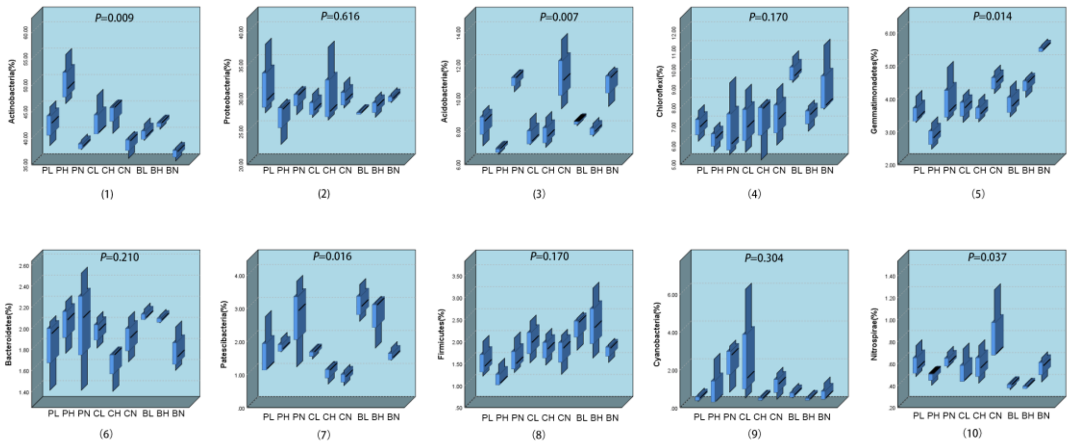

(B)

Figure S5. The relative abundance of the top 10 phyla as affected by different treatments. (A): Histograms of relative abundances. The horizontal axis represents the relative abundance (%), and asterisks indicate values that significantly differ in these treatments (Kruskal-Wallis H, * $p < 0.05$, **$p < 0.01$). (B) Box diagram of the dominant phylum. The significance (Kruskal-Wallis H) is marked at the top of the box diagram. P, C, and B represent hydroxyapatite addition, no amendments, and biochar addition, respectively. L, N, and H represent low planting density, no planting, and high planting density, respectively.

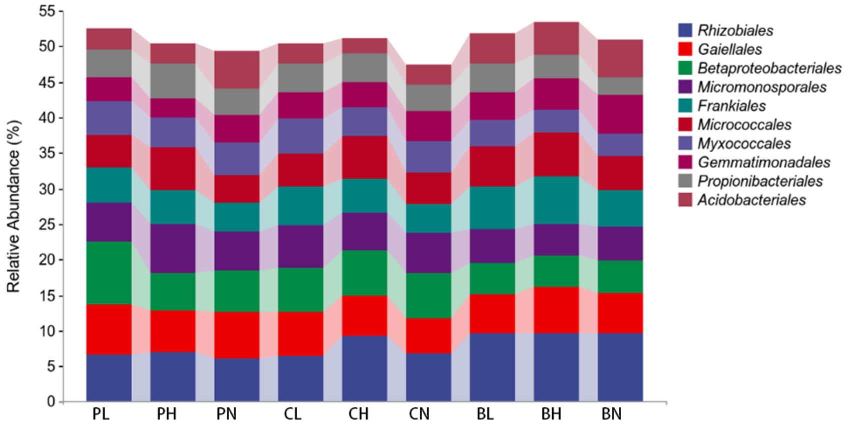

(A)

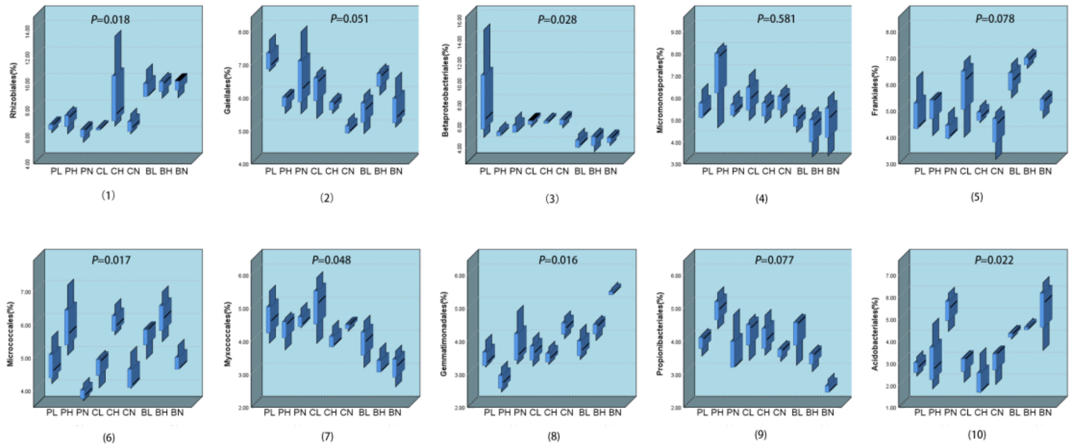

(B)

Figure S6. The relative abundance of the top 10 orders as affected by different treatments. (A): Histograms of relative abundances. (B): Box diagram of the dominant order. The significance (Kruskal-Wallis H) is marked at the top of the box diagram. P, C, and B represent hydroxyapatite addition, no amendments, and biochar addition, respectively. L, N, and H represent low planting density, no planting, and high planting density, respectively.

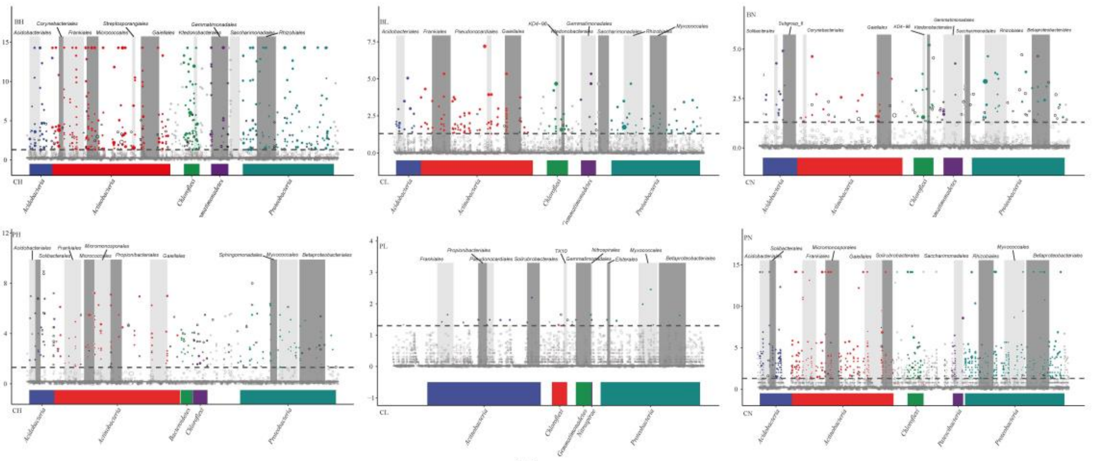

(A)

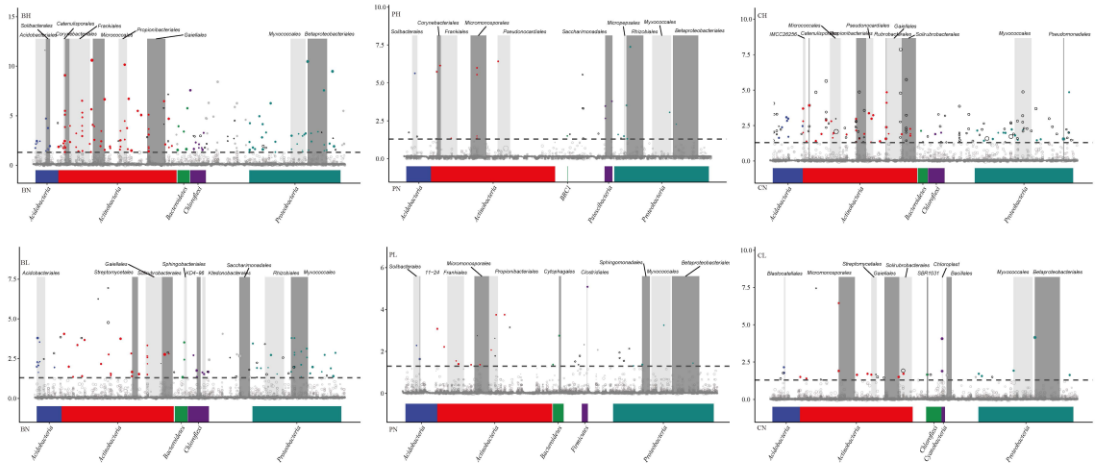

(B)

Figure S7. Manhattan plot of metagenomeSeq analysis. Significant differences were marked by colored dots or rings, non-significant differences were represented by gray rings. Significant differences were marked by colored dots or rings, non-significant differences were represented by gray rings. Each small image is labeled with control group at the lower left and up-regulated group at the upper left. Figure (A) shows differences in ASVs between SRA treatments (P, C, and B represent hydroxyapatite addition, no amendments, and biochar addition, respectively) and Figure (B) shows differences in ASVs between sweet sorghum cultivation treatments (L, N, and H represent low planting density, no planting, and high planting density, respectively.)

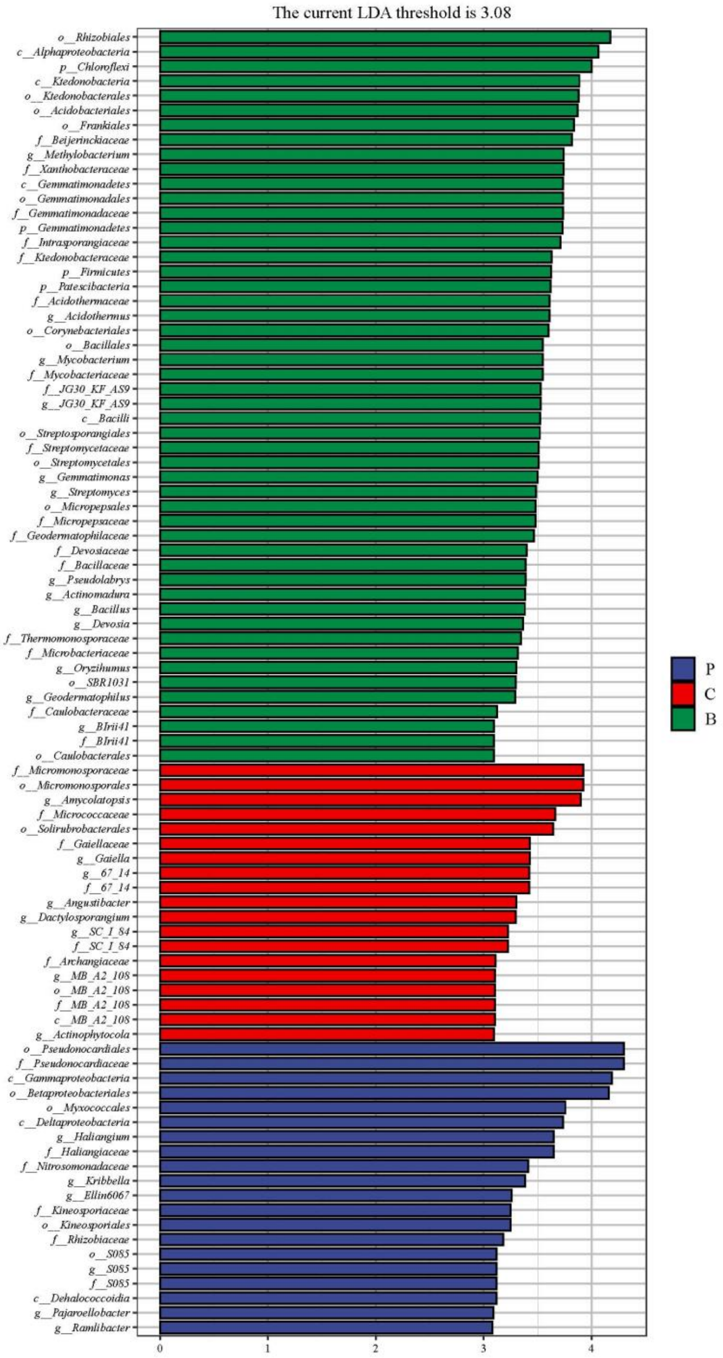

Figure S8. LDA scores for soil bacterial communities. The vertical coordinates show the significant differences between the groups, and the horizontal coordinates show the log LDA scores of the taxonomic units visually as a bar graph. Classification units are sorted according to their scores to depict their specificity in the sample grouping. The longer the length, the more significant the differences in the classification, and the color of the bar indicates the highest abundance sample grouping for that classification. Taxa enriched in B group were showed in green while taxa enriched in C group were shown in red and taxa enriched in P group were colored in blue. B treatment contains group BL, BH and BN; P treatment (HAP treatment) contains PL, PH and PN; C treatment (no SRA treatment) contains group CL, CH and CN.

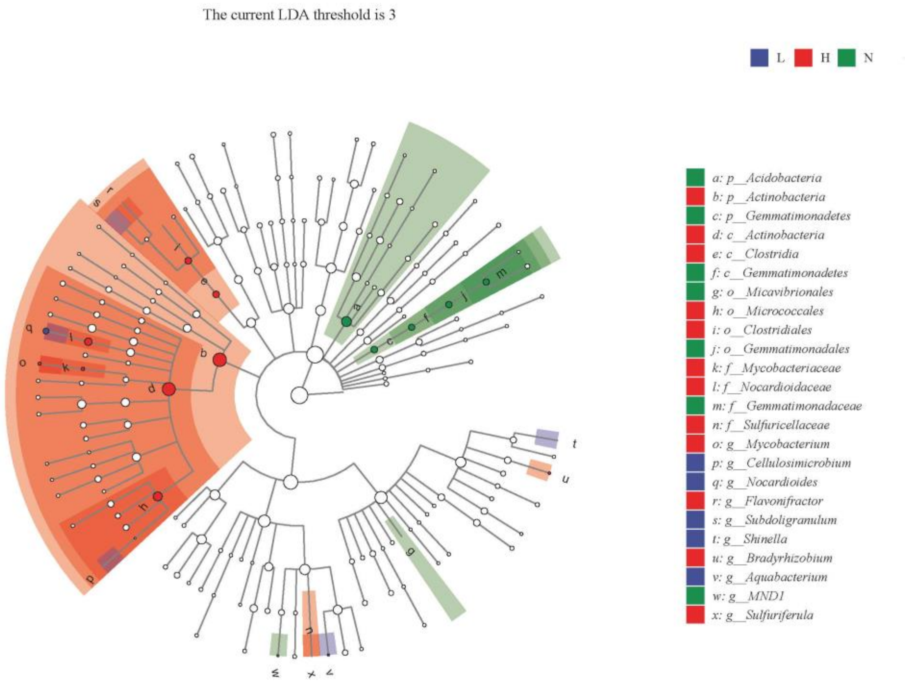

Figure S9. LEfSe taxonomic cladogram. The colored nodes from inner circle to outer circle represented the hierarchical elationship of all taxa from the phylum to the genus level. Taxa enriched in N group were showed in green while taxa enriched in H group were shown in red and taxa enriched in L group were colored in blue. Taxa with no significant difference between groups were hollow nodes, while these taxa with significant difference between groups were the nodes of other colors (bule, green and red). L, N, and H represent low planting density, no planting, and high planting density, respectively.

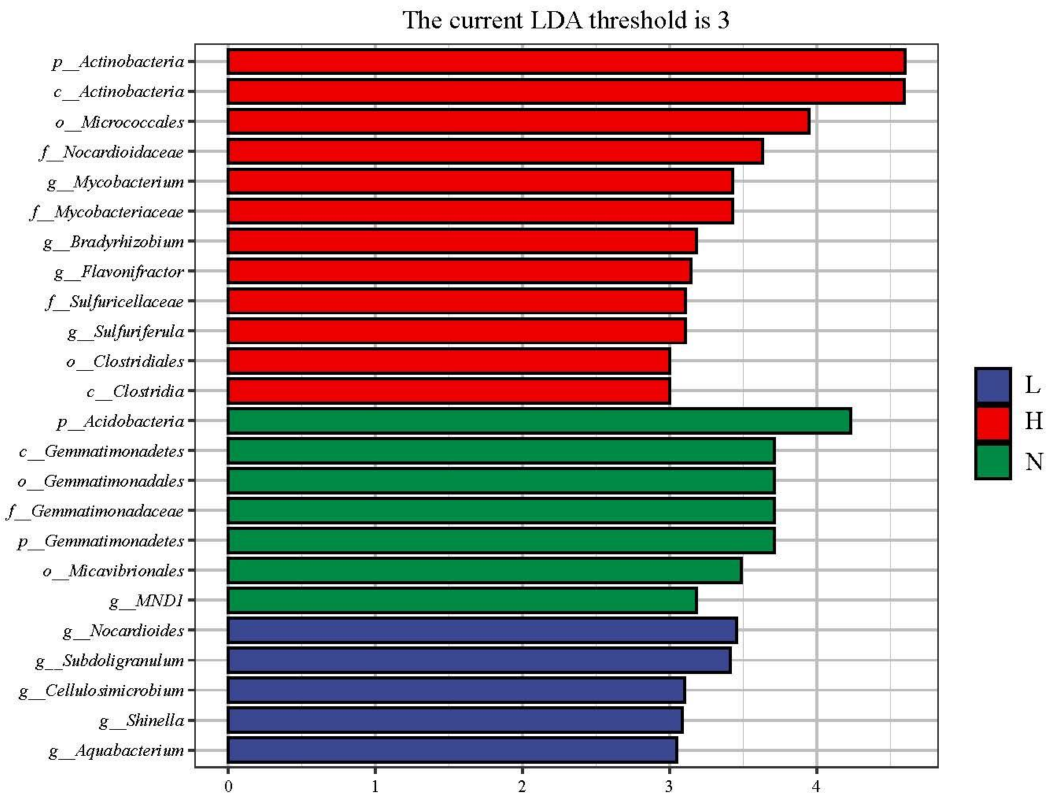

Figure S10. LDA scores for soil bacterial communities. The vertical coordinates show the significant differences between the groups, and the horizontal coordinates show the log LDA scores of the taxonomic units visually as a bar graph. Classification units are sorted according to their scores to depict their specificity in the sample grouping. The longer the length, the more significant the differences in the classification, and the color of the bar indicates the highest abundance sample grouping for that classification. Taxa enriched in N group were showed in green while taxa enriched in H group were shown in red and taxa enriched in L group were colored in blue. L, N, and H represent low planting density, no planting, and high planting density, respectively.
